# Macropinocytosis Exploitation by Cancers and Cancer Therapeutics

**DOI:** 10.3389/fphys.2016.00381

**Published:** 2016-09-12

**Authors:** Kevin D. Ha, Scott M. Bidlingmaier, Bin Liu

**Affiliations:** Department of Anesthesia, UCSF Helen Diller Family Comprehensive Cancer Center, University of California, San FranciscoSan Francisco, CA, USA

**Keywords:** macropinocytosis, receptor-dependent macropinocytosis, macropinosome, targeted cancer therapy, tumor selective internalization, altered membrane dynamics, neurodegenerative disease, pathogen entry

## Abstract

Macropinocytosis has long been known as a primary method for cellular intake of fluid-phase and membrane-bound bulk cargo. This review seeks to re-examine the latest studies to emphasize how cancers exploit macropinocytosis to further their tumorigenesis, including details in how macropinocytosis can be adapted to serve diverse functions. Furthermore, this review will also cover the latest endeavors in targeting macropinocytosis as an avenue for novel therapeutics.

## Introduction

Macropinocytosis is an endocytic pathway that leads to internalization of large patches of plasma membrane along with extracellular fluid through irregularly formed vesicles called macropinosomes. When compared to endosomes originating from coated vesicles, macropinosomes are significantly larger by a factor of up to a thousand-fold (Hansen and Nichols, 2009). Despite observations from as early as the 1930s that cancer cells exhibit hallmarks of macropinocytosis (Lewis, 1937), macropinocytosis was largely viewed as a canonical pathway for bulk-phase endocytosis. However, recent advances in research lead to surprising, direct roles for macropinocytosis within tumorigenesis. Furthermore, increasing numbers of studies demonstrated that macropinocytosis might play broader roles ranging from neurodegenerative disease to pathogen infiltration into host cells. This review aims to shed light on how the various aspects of macropinocytosis may be exploited by both cancers and anti-cancer therapeutics.

### The biomechanics of membrane ruffling and macropinocytosis

One of the hallmarks of macropinocytosis is its reliance on the formation of expansive membrane ruffles within the plasma membrane, which contrasts the canonical, endocytic pathways that depend on coat proteins such as clathrin and caveolin (Doherty and McMahon, 2009). The membrane ruffles facilitate formation of randomly sized vacuoles ranging from 0.2 to 5 μm in size, which are up to 50-fold larger than the average 0.1 μm size exhibited in protein-coated vesicles (Hewlett et al., 1994). Comprehensive reviews are available that cover the biomechanical underpinnings of macropinocytosis in detail (Kerr and Teasdale, 2009; Lim and Gleeson, 2011). Membrane ruffling is initiated by rapid polymerization of branching of actin filaments (Mullins et al., 1998) and direct or indirect disruption of actin dynamics impacts macropinocytosis (Seastone et al., 2001; Innocenti et al., 2004). Spatial regulation of macropinocytosis can be aided by actin motor proteins, possibly by delivering vesicles and proteins to requisite sites of membrane ruffling (Brzeska et al., 2016). Other spatial and temporal regulators include the Rho superfamily of GTPases, such as Rac and Cdc42, along with lipid rafts and lipid components such as phosphoinositides and cholesterol, phosphatidylinositol phosphates (PIPs), their accompanying kinases (PIKs) and phosphatases (Grimmer et al., 2002; Mañes et al., 2003; Innocenti et al., 2004; Redelman-Sidi et al., 2013; Levin et al., 2015; Viaud et al., 2016). The roles of both phospholipids and phosphoinositides in not only macropinocytosis but within general endocytosis have long been investigated, with numerous, comprehensive reviews available (Simonsen et al., 2001; Bohdanowicz and Grinstein, 2013). Lastly, distinct forms of membrane ruffling may occur. In addition to traditional membrane ruffling, another type, circular dorsal ruffles, exhibits a single, transient wave of membrane ruffles from the edges of the dorsal membrane that then constricts into an annulus within a short time scale, is capable of internalizing >50% of ligand-bound growth receptors within minutes (Orth et al., 2006). It remains unclear exactly why different forms of membrane ruffling occur and whether their ensuing macropinosomes obey similar biomechanics compared to macropinosomes originating from traditional forms of membrane ruffling.

Despite uncovering multiple proteins that partake in the signal transduction behind membrane ruffling, it remains unclear how membrane ruffling is regulated. Questions remain regarding how macropinocytosis can be specifically activated in response to certain stimuli within distinct cell populations. For example, macropinocytosis can have dramatically different effects on distinct cell types such as glioblastoma multiforme (GBM) and pancreatic cancer cells. When macropinocytosis is upregulated in GBM cells by a quinine-derivative chemical, Vacquinol-1, massive membrane ruffling and macropinocytosis lead to cell death through excessive vacuolization and deformations in the plasma membrane (Kitambi et al., 2014). On the other hand, pancreatic cancer cells develop increased macropinocytosis to enhance cancer progression (Commisso et al., 2013). Interestingly, Vacquinol-1 only induces massive macropinocytosis in GBM cells and not fibroblasts or neurons, implicating the need for specific protein players in macropinocytosis (Kitambi et al., 2014). These studies largely implicate an involvement of cell- and tissue-specific signal transduction pathways that may differentially regulate macropinocytosis to suit their differentiated functions. As discussed later in this review, tissue-specific effects on macropinocytosis can also be observed through modulation of various proteins. Current understanding of the biomechanics behind tissue-specific forms of macropinocytosis remains murky at best.

After the commencement of membrane ruffling, folds and buds of the plasma membrane can undergo fission to yield macropinosomes. This fission can be accommodated by the actin-associated protein, coronin, which helps form macropinocytic vesicles and dissociates shortly after vesicle fission from the plasma membrane (Hacker et al., 1997; BoseDasgupta et al., 2015). Depending on their origin and cell type, macropinosomes can either recycle back to the plasma membrane to recycle cell surface receptors or merge with the endosomal/lysosomal network (Schnatwinkel et al., 2004). The intracellular itineraries of macropinosomes may significantly overlap with clathrin-dependent, endocytic pathways, including mergers with early endosomes and the endosomal recycling complex (Donaldson et al., 2009). Multitudes of protein regulators define each step of intracellular vesicle trafficking and as a result, cell types with varying proteomic profiles exhibit diverse, intracellular trafficking functions. Nevertheless, it remains unclear how different cell types manage to achieve different, regulated forms of macropinocytosis and how they can specify the itineraries of macropinosomes.

### Confirming the involvement of macropinocytosis

As with other forms of endocytosis, macropinocytosis can be microscopically observed with fluorescent molecules. This method typically has allowed full observation of the entire macropinocytosis pathway, beginning with the formation of large endocytic vesicles at the cell surface and until endosomal maturation, where intracellular vesicles coalesce with the lysosomal network. Fluid-phase, soluble proteins and sugars such as albumin and dextran, respectively, have served as useful, fluorescent markers specific for macropinocytosis (Racoosin and Swanson, 1992; Li et al., 2013). These molecules can help implicate an involvement of macropinocytosis by specifically colocalizing with novel, endocytosed cargo. Endocytosed cargo that largely colocalize with either albumin or dextran, instead of coated-vesicle cargo such as transferrin or transferrin receptors, typically suggest macropinocytosis involvement. Furthermore, solutes and receptors that have been associated with macropinocytosis can potentially be used as macropinocytic markers themselves. Examples of this include immunofluorescence against macropinocytosis-dependent cell-surface receptors, such as EGFR or EphA2, which have both been used to induce or observe macropinocytosis (Berger et al., 2012; Ha et al., 2014). Not only can fluorescent microscopy decipher macropinocytosis *in vitro*, but it can be additionally employed for *in vivo* observation of macropinocytosis, including within xenograft tumors in mice (Commisso et al., 2014).

Colocalization with macropinocytosis markers alone is usually not enough to support macropinocytosis involvement. In addition to colocalization, studies typically utilize pharmacological reagents that have specific impacts on macropinocytosis but not canonical, protein-coated endocytosis. In this fashion, macropinocytosis-specific markers continue to be internalized into cells while markers for canonical protein-coated endocytosis, such as transferrin, suffer from disrupted internalization. Cargos of interest that continue to internalize into cells and colocalize with macropinocytosis-specific markers despite pharmacological inhibition of canonical forms of endocytosis strongly implicate an involvement for macropinocytosis. Amilorides, for example, have been shown to inhibit macropinocytosis while leaving other coat-dependent, endocytic pathways intact (West et al., 1989; Dowrick et al., 1993). Amiloride-based chemicals can inhibit Na^+^/H^+^ exchangers and consequently induce acidification at the plasma membrane, which interferes with the activation of Rac and Cdc42 GTPases and subsequent actin remodeling (Koivusalo et al., 2010). Chemicals that inhibit actin filament polymerization, such as cytochalasin D, also inhibit macropinocytosis (Schliwa, 1982; Heuser, 1989). Inhibitors that disrupt signal transduction pathways that regulate membrane ruffling, such as inhibitors against PI3K or PIP5K, also disrupt macropinocytosis (Araki et al., 1996; Brown et al., 2001). Other chemicals can activate macropinocytosis, such as aluminum fluoride, which activates ADP ribosylation factor 6 (ARF6)-dependent macropinocytosis (Radhakrishna et al., 1996). Other comprehensive reviews are available that dissect the broad arsenal of pharmacological reagents available to interrogate the numerous forms of endocytic pathways (Ivanov, 2008). Primary disadvantages are commonly associated with pharmacological reagents, however. These typically include unknown, cellular side effects that often reduce selectivity against specific forms of endocytosis.

In many cases, selective disruption of specific proteins may lead to fewer side effects in dissecting the multiple endocytic routes. Another review covers many protein and lipid regulators that can be overexpressed, ablated, or mutated to yield distinct effects on specific forms of endocytosis, including macropinocytosis (Amyere et al., 2002). Depending on the both the protein target and the severity of disruption, non-specific side effects can be somewhat controlled.

In summary, fluorescent microscopy, pharmacological disruption, and altered protein expression can all be employed to confirm specific involvement of macropinocytosis vs. canonical, endocytic pathways. The studies referenced throughout this review largely utilize combinations of these methods to suggest macropinocytosis involvement.

### Activation of macropinocytosis through receptors and ligands

Depending on the cell type, macropinocytosis can be primarily constitutive or induced. However, cells can commonly possess both forms of macropinocytosis. Furthermore, varying cell types can display wide ranges in macropinocytic activity. For example, macrophages and dendritic cells often utilize high levels of constitutive macropinocytosis to screen the extracellular environment for pathogenic materials (Kerr and Teasdale, 2009). On the other hand, neutrophils exhibit increased levels of macropinocytosis when sensing foreign pathogens, which might also aid in their ability to ingest pathogens (Lim and Gleeson, 2011).

Macropinocytosis is often mediated through stimulation of cell surface receptors. These include receptor tyrosine kinase (RTK) family receptors, where exosomes shed by cancer cells can internalize into distant cancer cells in a paracrine fashion through binding with RTKs (Li et al., 2010; Koumakpayi et al., 2011; Nakase et al., 2015), cell-surface proteoglycans which macropinocytose ligands containing closely spaced, basic amino acids such as lysine, arginine, and histidine (Magzoub et al., 2006), and G-protein coupled receptors (GPCRs). Podocytes and colorectal cancer cells both activate macropinocytosis through interactions between albumin-associated free fatty acids (FFAs) and GPCRs (Wu et al., 2013; Chung et al., 2015). Dendritic cells activate macropinocytosis through another GPCR, S1P receptors, to detect S1P and activate macropinocytosis (Ocaña-Morgner et al., 2011). Certain cell types, including murine bone marrow macrophages, do not require macropinocytosis stimulation and instead have constitutive macropinocytosis (Norbury et al., 1995).

Pathogens commonly display surface glycans to trigger macropinocytosis through interactions with C-type lectin receptors on antigen-presenting cells (Frenz et al., 2015). In other instances, certain receptors bind to non-protein ligands to activate macropinocytosis. For example, calcium is necessary for inducing constitutive macropinocytosis in cells through G-protein-coupled calcium-sensing receptors (Canton et al., 2016). RTKs can also activate macropinocytosis through downstream activation of a small GTPase, Rab5, to induce circular membrane ruffles (Lanzetti et al., 2004).

Studies have demonstrated that proteins rich in arginine amino acids may internalize into cells specifically via macropinocytosis, possibly through basic interactions that results in crosslinking (Nakase et al., 2004). Indeed, a 12-mer arginine peptide, but not an 8-mer, induces macropinocytosis by cross-linking CXCR4, the co-receptor for HIV-1 infection into host cells (Tanaka et al., 2012). Additionally, cross-linking the complement receptor, CD46, either via antibodies or through pathogens such as the measles virus, induces macropinocytosis (Crimeen-Irwin et al., 2003). Receptor cross-linking and macropinocytosis activation may be more generic than previously thought, as studies have shown that receptor cross-linking enhances endocytosis (Moody et al., 2015).

### Cancers exploit macropinocytosis to enhance tumorigenesis

Numerous studies have firmly established concrete roles for Rac in broad aspects of tumorigenesis, including cell survival and growth, metastasis, and secondary tumor establishment (Mack et al., 2011). Although Rac plays an important role as an upstream activator of macropinocytosis, it is difficult to determine whether macropinocytosis itself plays direct roles in the tumorigenic functions of Rac. Rac can be activated by members of the Ras (rat sarcoma) GTPase superfamily, which has long been heralded as the most frequently mutated family of genes in cancer (Stephen et al., 2014). Certain proteins, such as Abi1, can regulate both macropinocytosis and protein coat-dependent endocytosis and therefore it is likely that cancer cells can exploit multiple endocytic pathways to establish and maintain their oncogenic phenotypes (Innocenti et al., 2005).

Macropinocytosis serves as an efficient method to internalize cell surface receptors, which cancer cells can exploit in their favor. Cancers down-regulate cell-surface death receptors (DRs) via macropinocytosis in an H-Ras dependent manner to evade TNF-related apoptosis-inducing ligands (TRAILs) and subsequent apoptosis (Chen et al., 2014). Interestingly, certain cancers with either K- or H-Ras mutations can still be sensitive to TRAILs as other receptors and downstream signaling components may affect DR internalization via macropinocytosis (Drosopoulos et al., 2005; Wang et al., 2015). Interestingly, cancers can utilize macropinocytosis to internalize and activate receptors. Prostate and breast cancer cells utilize macropinocytosis to translocate a growth factor receptor, ErbB3, from the plasma membrane into the nucleus to further cellular proliferation (Koumakpayi et al., 2011; Reif et al., 2016). It remains unclear exactly how a membrane-bound receptor, possibly within a vesicle, is imported into the nucleus. Additionally, H-Ras-transformed fibroblasts internalize cell-surface platelet-derived growth factor β-receptors (PDGFRβ) through macropinocytosis to sensitize and enhance PDGFRβ activation, resulting in increased anchorage-independent proliferation (Schmees et al., 2012). In this case, PDFGFRβ phosphorylation is enhanced only within macropinosomes, possibly due to macropinosomes-specific PI3K activity (Schmees et al., 2012).

As macropinocytosis is an efficient and rapid form of endocytosis, it is not surprising that cancers have exploited it to replenish scarce nutrients for sustained propagation within the tumor microenvironment. Ras-transformed pancreatic cancer cells can upregulate macropinocytosis to internalize and degrade albumin as a source of glutamine, which is one of the most deprived metabolites within tumor microenvironments (Commisso et al., 2013; Kamphorst et al., 2015). Macropinocytosis appears to be the key player in this phenomenon, as amiloride-based drugs that selectively inhibit macropinocytosis while leaving other coat-dependent endocytosis intact halted intake of albumin as a source of glutamine for cancer cells (Commisso et al., 2013). In a similar fashion to absorbing extracellular albumin, cancer cells have also been shown to internalize extracellular ATP to aid in cancer metabolism (Qian et al., 2014).

In addition to accumulating fluid-phase nutrients such as proteins and ATP, cancer cells often internalize secreted vesicles, called exosomes or microvesicles, through macropinocytosis (Nakase et al., 2015). Exosomes can carry proteins, lipids, and nucleic acids to serve as a form of extracellular communication or metabolic replenishment (Tkach and Thery, 2016). Many cancer types commonly exploit exosomes to enhance cancer progression, typically by secreting exosomes that internalize into other cancer cells and lead to favorable environments that promote angiogenesis, metastasis, and immunosuppression (Whiteside, 2016). For pancreatic cancer cells, internalizing exosomes requires K-Ras and EGFR-dependent macropinocytosis (Nakase et al., 2015). Alternatively, exosomes originating from cancer cells can cause dysfunction in normal cells. For example, pancreatic cancer cells shed exosomes that can be internalized by β-cells to negatively impact insulin secretion and further pancreatic cancers (Javeed et al., 2015). Other cell types, such as cancer-associated fibroblasts, do not require oncogenic K-Ras signaling to internalize exosomes (Zhao et al., 2016).

### Disrupting macropinocytic biochemistry for therapeutic gains

Established links between macropinocytosis and cancers have spurred the development of therapeutics targeting the biochemical regulation behind macropinocytosis. This includes therapeutics against the phosphoinositide biochemical pathways. For example, RNA interference (RNAi) of one particular PIP5K, Iγi2, leads to decreased oncogenic growth of breast cancer cells (Thapa et al., 2013). In this case, Iγi2 coordinates with Src to promote anchorage-independent growth. PIP5K may serve divergent functions across multiple tissue types. For example, disruption of PIP5Kα stunted membrane and protein recycling from macropinosomes to the plasma membrane (Brown et al., 2001). It remains unclear whether membrane and protein recycling from macropinosomes contributes to anchorage-independent growth in breast cancer cells. In another example, the fungal metabolite wortmannin can block phosphoinositide-3-kinase (PI3K) to inhibit scission of macropinosomes from the cell surface, thus negatively effecting pancreatic cancer motility, invasion, and metastasis (Araki et al., 1996; Teranishi et al., 2009). Future studies investigating overlapping functions across various phosphoinositide pathways within the context of macropinocytosis across multiple cancer cell types can potentially uncover additional therapeutic opportunities.

Another therapeutic venture includes disrupting cancer cell metabolic activity through inhibition of macropinocytosis (Zeitouni et al., 2016). However, cancer cells may additionally employ other compensatory methods such as membrane transporters and coat-dependent endocytosis to import extracellular nutrients and proteins (Selwan et al., 2016). Indeed, macropinocytosis can activate mTORC1 through nutrient-intake in cancer cells that rely on metabolizing endocytosed proteins in scarce amino-acid conditions (Palm et al., 2015; Yoshida et al., 2015). Macropinosomes eventually mature into increasingly acidic vacuoles, or lysosomes, for subsequent degradation of consumed proteins (Racoosin and Swanson, 1993). Hydroxychloroquine (HCQ), which inhibits lysosomal acidification, negatively impacts both autophagy and macropinocytosis-dependent scavenging and is currently under investigation for its cancer therapeutic potential (Wolpin et al., 2014). In this context, HCQ can be used to prevent cancer cells from breaking down extracellular proteins to generate metabolic substrates, thereby placing limitations on cancer cell metabolism and growth (Kimmelman, 2015).

Lastly, other therapeutic ventures have shown that hyper-stimulating macropinocytosis in cancer cells can lead to non-apoptotic cell death known as methuosis (Li et al., 2010; Maltese and Overmeyer, 2015). Methuosis can be induced through hyper-activated Ras in glioblastoma cells, contributing to excessively large macropinosomes that ultimately result in cell rupture (Overmeyer et al., 2008). As Ras is commonly upregulated in numerous cancers, glioblastoma cells must lack certain signal transduction pathways that regulate macropinocytosis levels. Uncovering these pathways, if they exist, should help expand the arsenal of available targets in disrupting macropinocytosis.

### Exploiting macropinocytosis for targeted delivery of anti-cancer therapeutics

As cancer cells frequently employ macropinocytosis to aid in receptor regulation and internalize essential metabolites, extensive efforts have been underway in utilizing macropinocytosis to deliver cytotoxic therapeutics specifically into cancer cells. Some anti-cancer agents innately undergo macropinocytosis, such as AS1411, which internalizes into various cancer cells through cell-surface nucleolin-dependent mechanisms that activate macropinocytosis only in malignant cells (Reyes-Reyes et al., 2010). Other therapeutics specifically target cell surface receptors that may trigger macropinocytosis. For example, therapeutic drugs conjugated with peptides that target a combination of proteoglycans and keratinocyte growth factor receptors (KGFR) can selectively internalize into and kill KGFR-expressing lung cancer cells via macropinocytosis (Iglesias and Koria, 2015).

Potent cancer therapeutics that exhibit excessive, systemic toxicities or unstable pharmacokinetics from hydrophobic profiles are excellent candidates for conjugation to yield chemical conjugates or nanoparticles. The resultant conjugates or nanoparticles can be engineered to yield greater specificity and enhanced pharmacokinetics. In many other examples, synthetic conjugates comprising any combination of small chemicals, lipids, proteins, genetic components, and chemical scaffolds can be developed to form nanoparticles that can then be internalized into target cells via macropinocytosis. Nanoparticles, which vary widely in composition, size, chemical charge, and shape, all contribute to distinct, cellular specificities and in endocytic mechanisms (Kettler et al., 2014). Larger nanoparticles rely on macropinocytosis for efficient internalization but smaller nanoparticles can traffic into cells through protein-coated endocytosis as well. For example, nanoparticles featuring paclitaxel fused to a chimeric peptide comprising an elastin-like peptide with a hydrophilic peptide (CP-PTX) serve double purposes in reducing the hydrophobic profile of paclitaxel and in enhancing tumorigenic internalization of paclitaxel via macropinocytosis (Bhattacharyya et al., 2015; Iglesias and Koria, 2015). Nanoparticles can also deliver genetic materials. For example, polyethylene glycol (PEG)-based nanoparticles were successfully used to deliver genes into cancer cells through macropinocytosis (Walsh et al., 2006). Nanoparticle shapes play important roles as well, as rod-like nanoparticles internalize into cells independently from macropinocytosis (Liu et al., 2016). Nanoparticles can also be targeted against specific cell types by incorporating aptamers into lipid nanoparticles (Liang et al., 2015). Similarly, nanoparticles conjugated with antibodies targeting the collagen receptor, α2β1 integrin, lead to efficient internalization via macropinocytosis (Kankaanpää et al., 2015). As α2β1 integrins have been previously implicated in both cancer stem cells and tumor angiogenesis, α2β1 integrin-targeting nanoparticles could yield specific utility in cancer therapeutics (Naci et al., 2015).

Another intriguing therapeutic front includes conjugating cytotoxic payloads onto albumin primarily for enhancing drug pharmacokinetics and because albumin has long been observed to accumulate within solid tumors through macropinocytosis (Kratz, 2008). An example includes the FDA-approved, nanoparticle albumin-bound form of paclitaxel (nab-paclitaxel or Abraxane®) for treating multiple cancers. However, cancers are still able to overcome these drugs through acquired resistance, likely in differentially expressed proteins that regulate macropinocytosis, including cytoskeletal and lipid metabolism proteins (Zhao et al., 2015b) or through increased drug exporters such as P-glycoprotein (Zhao et al., 2015a). Other albumin-based conjugates targeting folate receptors also demonstrated efficient delivery of cytotoxic compounds specifically into cancer cells (Shi et al., 2014). Conjugates with non-albumin carriers have also been successful, including paclitaxel poliglumex (PPX or formerly Xyotax), comprising paclitaxel and polyglutamic acid polymers. PPX has been effective against metastatic breast cancers when used in combination with capecitabine (Northfelt et al., 2014).

Another component commonly used for conjugation is the poly-arginine peptide. As poly-arginine peptides induce macropinocytosis and cancer cells generally have increased macropinocytosis, efforts have been underway in conjugating poly-arginine peptides with cytotoxic compounds. These conjugates can be employed to deliver a variety of materials into cells, including cytotoxic reagents against cancer cells (Biswas et al., 2013; Liu et al., 2014), genetic materials (Zhang et al., 2006; Hayashi et al., 2012), insulin (Liu et al., 2012; Zhang et al., 2015), and liposomes across the blood brain barrier (Qin et al., 2012). Interestingly, at least in HeLa cancer cells, poly-arginine peptide entry into the cell cytosol occurs independently from macropinocytosis (Zaro et al., 2006). This suggests that poly-arginine peptides endocytosis may not be exclusive to macropinocytosis, depending on both the cargo and cell type.

As cancers routinely internalize exosomes, a logical maneuver includes formulating therapeutics that mimic exosomes. Exosomes can be derived from same-host cells to avoid inducing immune responses (Hall et al., 2016). Alternatively, exosomes originating from dendritic cells may help elicit immune responses by T and NK cells to specifically target cancer cells (Pitt et al., 2016). Exosomes can be prepared *in vitro*, with therapeutic proteins either electroporated into exosomes or intracellularly incorporated through targeted overexpression of genes in the cell cultures (Munson and Shukla, 2015). Furthermore, lipid-membrane coat-based delivery systems that resemble exosomes can be engineered in ways that promote endosomal escape after macropinocytic entry into cells to enable gene delivery into cells while avoiding degradation through the lysosomal pathway (Khalil et al., 2007). Exosomes are extremely useful in delivering payloads into cancer cells without requiring advanced chemical conjugation as often required in nanoparticles. Additionally, exosomes also offer greater flexibility in packaging diverse combinations of cargoes.

### Macropinocytosis in pathogen-host interaction and neurodegenerative diseases

If cancers commonly exploit macropinocytosis for efficient endocytosis of scarce nutrients and proteins, it is not surprising that various pathogens also infiltrate into host cells through this pathway. Pathogens such as viruses and bacteria commonly enter human cells by activating macropinocytosis through receptor-dependent means. Several examples include human cytolomegavirus (Hetzenecker et al., 2016), *Mycobacterium smegmatis* (Baltierra-Uribe et al., 2014), influenza A virus (de Vries et al., 2011), vaccinia virus (Mercer and Helenius, 2008), infectious bursal disease virus (Gimenez et al., 2015), *Salmonella* (Francis et al., 1993; Chen et al., 1996), Zaire Ebola virus (Hunt et al., 2011), and Kaposi's sarcoma-associated herpesvirus (Chakraborty et al., 2012). However, pathogens may enter cells through additional endocytic routes in addition to macropinocytosis. For example, African swine fever virus can internalize into macrophages through either macropinocytosis or clathrin-mediated endocytosis (Hernáez et al., 2016). Organisms that are larger than bacteria and viruses, such as protozoa, can also infiltrate into host cells through macropinocytosis (de Carvalho et al., 2015). In addition, *Plasmodium* sporozoites, or malaria, also infiltrate into host hepatocytes by inducing macropinocytosis through the EphA2 receptor (Kaushansky et al., 2015), possibly through receptor cross-linking (Ha et al., 2014).

Macropinocytosis appears to play vital roles across a variety of neurodegenerative diseases as well, and this has been comprehensively reviewed (Zeineddine and Yerbury, 2015). These diseases include Parkinson's disease, Huntington's disease, amyotrophic lateral sclerosis, and Alzheimer's disease. Macropinocytosis allows neuronal cells to internalize protein aggregates, possibly through cross-linking receptors that remain largely unknown. Cross-linking of amyloid precursor protein (APP) at the cell surface by antibodies leads to APP internalization via Arf6-depedent macropinocytosis, potentially contributing to Alzheimer's disease (Tang et al., 2015). In some of these cases, heparin sulfate proteoglycans may serve as the receptors for internalizing tau and α-synuclein fibrils to commence intracellular fibrillization (Holmes et al., 2013). As neurodegenerative fibrils commonly utilize macropinocytosis to infiltrate neuronal cells, therapeutics that specifically inhibit macropinocytosis in neurons sound logically promising. However, such therapeutics are currently not available due to the lack of knowledge in the signal transduction pathways that define both tissue-specific and universal aspects of macropinocytosis regulation. For example, therapeutics that exhibit strong, inhibitory effects against macropinocytosis may yield unintended side effects in disrupting constitutive macropinocytosis of both macrophages and dendritic cells.

## Conclusions

**Figure 1:**
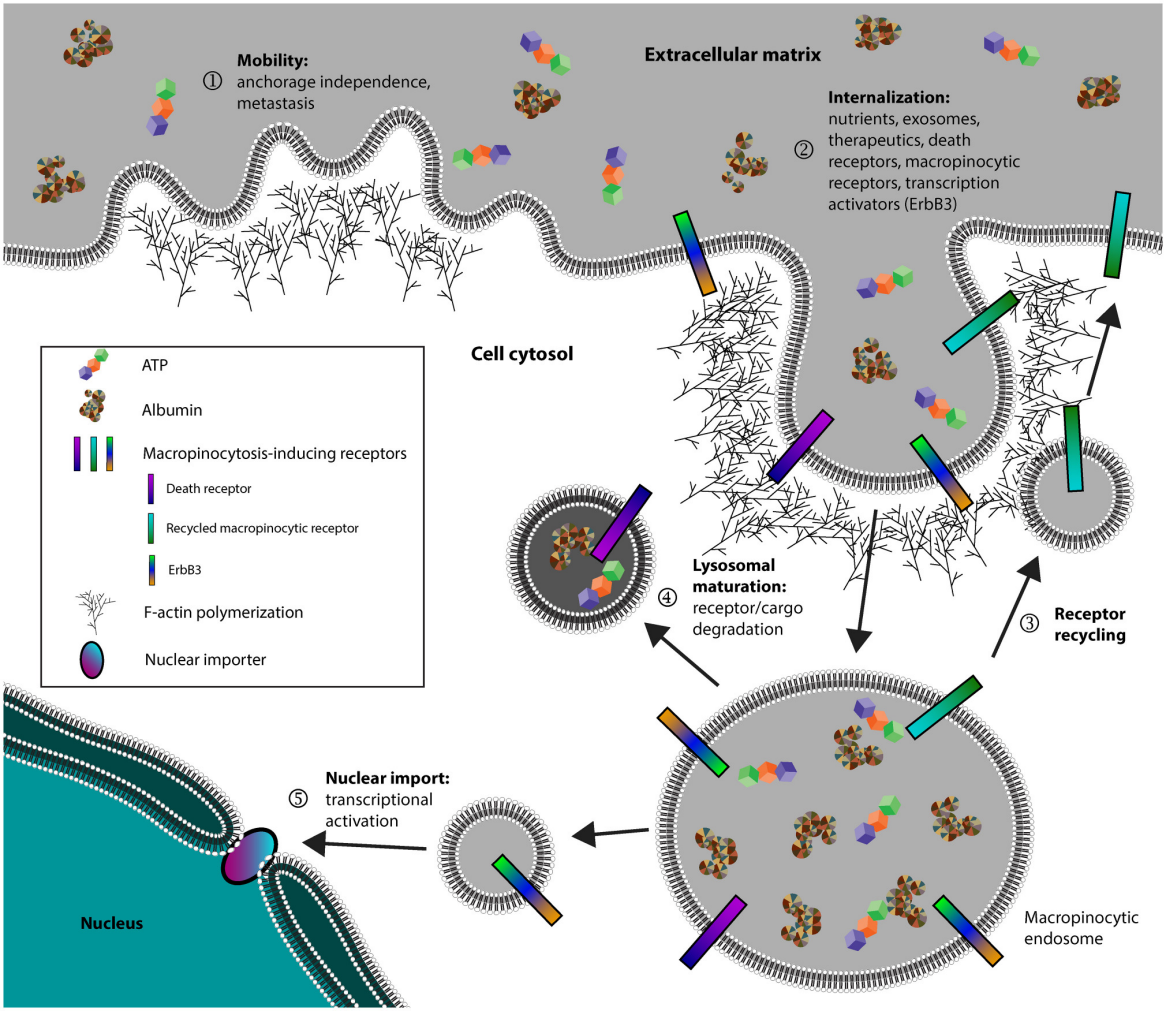
**Cancer cells exploit macropinocytosis to fulfill diverse functions**. (1) Cancer cells can utilize membrane ruffles, a hallmark of macropinocytosis, to establish mobility and facilitate both anchorage independence and metastasis. (2) Cancer cells internalize metabolic nutrients, surface receptors, and transcription activators. (3) Receptors involved in activating macropinocytosis are recycled back to the plasma membrane. (4) Non-recycled protein cargo and receptors are degraded through the lysosomal network or in some instances, (5) certain receptors such as ErbB3 can be targeted for nuclear import to activate transcription.

Cancer cells, in addition to pathogens and neurodegenerative diseases, all exploit macropinocytosis for its efficiency in endocytosis. In particular, cancer cells seem to possess increased macropinocytic activity to fulfill diverse functions that include metastasis, metabolism, and signal transduction (Figure 1). For this reason, there has been an increased interest in the field of macropinocytosis-dependent therapeutics, expanding the available arsenal in selectively combating cancers. Additional studies in how different cancers utilize macropinocytosis to further disease progression could allow development of novel therapeutics against specific cancers. On this same note, it will be interesting to determine how macropinocytosis is differentially regulated across various tissues and cell types. Understanding how specific cell types can uniquely regulate macropinocytosis may pave way for novel therapeutics that can specifically target those cell types. Lastly, further research in how macropinocytosis is uniquely regulated from other endocytic pathways should facilitate targeted therapeutics without lending systemic toxicity stemming from broadly inhibiting all endocytic pathways.

## Author contributions

KH wrote the manuscript, SB edited the manuscript, BL selected the review topic and edited the manuscript.

### Conflict of interest statement

The authors declare that the research was conducted in the absence of any commercial or financial relationships that could be construed as a potential conflict of interest.
